# A Novel Polymer Insect Repellent Conjugate for Extended Release and Decreased Skin Permeation of Para-Menthane-3,8-Diol

**DOI:** 10.3390/pharmaceutics13030403

**Published:** 2021-03-18

**Authors:** Sayyed I. Shah, Vitaliy V. Khutoryanskiy, Adrian C. Williams

**Affiliations:** Reading School of Pharmacy, University of Reading, Whiteknights, Reading RG6 6AD, UK; S.I.Shah@pgr.reading.ac.uk (S.I.S.); v.khutoryanskiy@reading.ac.uk (V.V.K.)

**Keywords:** polymer–drug conjugate, extended release, skin, insect repellent, p-menthane-3,8-diol, PMD

## Abstract

Background: We developed a novel polymer insect repellent conjugate for extended release and decreased skin permeation of the volatile insect repellent p-menthane-3,8-diol (PMD). Methods: PMD was conjugated with acryloyl chloride via an ester bond to form acryloyl–PMD, which was subsequently copolymerised with acrylic acid at varying molar ratios. Copolymer structures were characterised by ^1^H NMR and FT-IR, analysed by thermogravimetric analysis (TGA), differential scanning calorimetry (DSC), molecular weights and reactivity ratios determined, and repellent loading assessed. Results: Using porcine liver esterases, ~45% of the insect repellent was released over five days. Penetration and permeation studies of the copolymer and free repellent using excised, full-thickness porcine ear skin showed no detectable permeation of the copolymer through skin compared to the PMD. Moreover, tape stripping revealed that over 90% of the copolymer remained on the outer surface of the skin, whereas free PMD was within all skin layers. A planarian toxicity fluorescence assay indicated that that the copolymer is unlikely to be a significant irritant when applied topically. Conclusions: this study demonstrates the feasibility of the copolymer approach to develop extended-release insect repellents while reducing skin uptake and transdermal permeation of the small-molecular-weight active ingredient, in order to minimise any adverse effects.

## 1. Introduction

Insect repellents are widely applied to prevent bites and associated inflammation or rash, and as a protective measure against insect-borne diseases, including malaria, dengue fever, and leishmaniasis [1,2]. Numerous insect repellents are available, including p-menthane-3,8-diol (PMD or menthoglycol), which is naturally derived from eucalyptus plants and is used commercially. However, PMD is highly volatile, and adverse effects associated with topical absorption, notably in pregnant women and infants, have been reported [2].

Human skin provides a remarkable self-repairing barrier to the ingress of exogenous chemicals [3]. However, it also provides a well-established route for drug delivery from topical formulations for both local (for example, hydrocortisone cream) and systemic (for example, from transdermal patches) actions. In general, molecules that can effectively penetrate into and permeate through intact human skin tend to be relatively low molecular weight (<~500 Da) and lipophilic (log *P*_(octanol/water)_ = ~1–4). While multiple factors can affect the absorption of chemicals into or through skin, the transdermal flux of the repellent *N*,*N*-diethyl-meta-toluamide (DEET) through porcine skin has been reported to be in the region of 2 µg/cm^2^/h [4], whereas PMD has been purported to act as a skin penetration enhancer, increasing the delivery of antipyrine and indomethacin through pig skin [5]. Insect repellents are generally volatile materials that form a surface “cloud” to repel biting insects. Consequently, repeated dosing is required, which can also increase uptake into and permeation through the skin. To mitigate this, polymeric formulations have been explored [6]. For example, polymeric micro- or nano-capsules have been developed to encapsulate insect repellents, such as DEET, PMD, citronella oil, picaridin, and others [7,8,9,10]. Typically these approaches extend the duration of efficacy up to a maximum of 24 h (for PMD), but increased skin residence from formulations with greater substantivity may further increase the permeability of the repellent through the skin.

Here we have synthesised a polymer–repellent conjugate through an ester bond with PMD. The skin is metabolically active and possesses esterases, albeit at lower levels (~10%) than the liver. These enzymes have previously been used to transform a drug–drug conjugate to aid in transdermal delivery [11]. By conjugating a small molecule repellent to a relatively large molecular weight polymer, we hypothesised that drug uptake into the skin will be minimised, with the repellent released in a sustained manner over extended periods of time. After conjugating the PMD with acryloyl chloride via an ester bond to form acryloyl–PMD (APMD), the conjugated monomer was copolymerised with acrylic acid (AA) to form a series of copolymers, poly(AA–*co*–APMD). Reagents and copolymers were characterised and the thermal stability of the repellent-containing copolymer was assessed. Release testing of the repellent from copolymers used an in vitro porcine liver esterase (PLE) assay, and the ability of the copolymer to reduce PMD uptake into and permeation through the skin was assessed in penetration and permeation studies using pig ear skin.

## 2. Materials and Methods

### 2.1. Materials

PMD (p-menthane-3,8-diol) was used as received (BOC, New York, NY, United States). D-Squame tapes were obtained from Clinical & Derm (Des Plaines, IL, United States). Parafilm M was obtained from AMCOR (Chino, CA, United States). Acrylic acid (AA) in liquid form (99%, Fisher Scientific, Loughborough, United Kingdom) was passed through the aluminium oxide column to remove the inhibitor prior to use in copolymerization. Azobisisobutyronitrile (AIBN) was used as an initiator, and was recrystallized twice in methanol (Sigma-Aldrich, Gillingham, United Kingdom). Sodium hydroxide (Fisher Scientific, United Kingdom), acryloyl chloride, and anhydrous triethylamine (Sigma-Aldrich, United Kingdom) were used as received. Porcine liver esterases (PLEs), acryloyl chloride, and ammonium acetate were from Sigma-Aldrich (Merck, United Kingdom). Poly(acrylic acid) (weight–average molecular weight (Mw) = 6000 Da) was purchased from Polysciences (Bergstrae, Germany). Acetonitrile, ethanol and all the solvents used were of liquid chromatography–mass spectroscopy (LC–MS) grade and were procured from Fisher Scientific (United Kingdom), and were used without further purification. Thin-layer chromatography (TLC) plates were from Merck (Darmstadt, Germany).

### 2.2. Methods

#### 2.2.1. Synthesis of Monomer–PMD Conjugate

A total of 3.54 g of PMD was dissolved in 4 mL of anhydrous tetrahydrofuran (THF) and stirred for 15 min until a clear solution was obtained. Then, 3.44 mL of anhydrous triethylamine was added to de-protonate the PMD, and was allowed to stir for 5 min. To this 1.8 mL of acryloyl chloride was added dropwise. As this is a highly exothermic reaction, the round-bottom flask was placed over ice. The mixture was stirred at room temperature for 4 h before the solvent was evaporated under a vacuum, followed by silica column chromatography using mixtures of ethyl acetate and hexane (1:4 *v/v*) to select product from the secondary alcohol, verified by thin-layer chromatography (TLC) and NMR data. The final product was a viscous oil (2.1 g, yield = 53%).

^1^H NMR, (DMSO, 400 MHz): δ 6.40 ppm (1H, d), 6.25 ppm (1H, q), 5.90–6.00 ppm (1H, d), 5.30 ppm (1H, s), 4.20 ppm (1H, s), 0.90 ppm (6H, s), 0.80 ppm (3H, d). IR data: 3221 cm^−1^ = OH of PMD; 2840–2970 cm^−1^ = C–H stretches; 1719 cm^−1^ = ester linkage; 1017–1048 cm^−1^ = C–H bending, *m*/*z* = 226.3 Da, which corresponds to C_13_H_22_O_3_.

#### 2.2.2. Synthesis of Copolymers

Poly(AA-*co*-APMD) was synthesised via free radical copolymerisation with varying molar ratios in the feed mixture. Briefly, in a typical reaction (AA/APMD = 30:70), APMD (300 mg), acrylic acid (285 mg), and azobisisobutyronitrile (AIBN; 0.005 mol/L) were added into ethanol (5 mL). After degassing the solution with nitrogen for 20 min, the mixture was stirred under the inert environment at 65 °C for 16 h. Then the flask was cooled to quench the reaction, and the obtained product was dialysed using a regenerated cellulose membrane (Sigma Aldrich, United States; MWCO 2000 Da) against ethanol for 2 days, with six changes of solvent to remove unreacted monomer or oligomers. The final product poly(AA–*co*–APMD) was obtained using a rotary vacuum evaporator for 20–30 min at 50 °C. The yield of the copolymer after the purification was 18–50% for different molar ratios, except AA/APMD (10:90), where the yield was extremely low (2%), and attempts to obtain a homopolymer of APMD failed.

#### 2.2.3. Molecular Weight and Molecular Weight Distribution of the Copolymers

The molecular weight of the copolymers and its distribution was determined using gel permeation chromatography (GPC) with an Agilent Technologies 1260 Infinity systemsm with a pore size of 250, 500, and 1000 Å. The mobile phase was THF at a flow rate of 0.75 mL/min. The eluent was dimethylformamide (DMF) containing Bu_4_NBr (0.1% *w/v*), and polystyrene standards were used for calibration.

#### 2.2.4. Reactivity Ratio Determination

Monomer mixtures of AA and APMD were prepared in 5 mL of ethanol at five different molar ratios (AA/APMD = 10:90, 30:70, 50:50, 70:30, 90:10) with the AIBN concentration kept constant in all cases (0.005 mol/L). Polymerisation, at 60 °C, was allowed to proceed to low conversions (below 10%), and so reactions were terminated before 2 h. Unreacted AA and APMD was then quantified using liquid chromatography–mass spectroscopy (LC–MS), from which the composition of the copolymers could be determined.

#### 2.2.5. Thermal Analysis

Thermogravimetric analysis (TGA) of copolymers and reagents used a Q50 thermogravimetric analyser (TA Instruments, Crawley, United Kingdom) purged with nitrogen to provide an inert environment. Samples were placed in an aluminium pan and then on a platinum TGA pan, and thermal decomposition was assessed; for copolymers, this was from 35 to 600 °C, whereas for the volatile PMD temperature ramped up to 100 °C, in all cases at 5 °C/min.

Differential scanning calorimetry (DSC) used a DSC Q1000 (TA Instrument, United Kingdom) calibrated with an indium standard. Samples (4–10 mg) were accurately weighed and placed in a hermetically-sealed aluminium pan with a pin hole. The samples were then subjected to hot/cool/hot cycles, under a constant flow of nitrogen at 50 mL/min, with heating and cooling rates of 10 or 50 °C/min, respectively. Glass transition temperatures were determined from the second heating cycle. All thermal analyses were performed in triplicate.

#### 2.2.6. In Vitro Hydrolysis of the Copolymer by Porcine Liver Esterases (PLEs)

The AA/APMD (30:70) copolymer was selected for these studies. Samples (10 mL) were prepared at 5 mg/mL in water with acetonitrile (9:1 *v/v*), and the pH adjusted to 7 by ammonium acetate buffer. Then, 35 mg of PLEs (1 unit of enzyme per µM of the conjugate) was added, and the solution stirred at 32 ± 1 °C to mimic external skin temperature. Samples (0.75 mL) were periodically withdrawn and quenched with an equivolume of acetonitrile (0.75 mL) before centrifugation (Sanyo MSE Micro Centaur MSB010.CX2.5) at 13,000 rpm for 12 min; the supernatant was collected and analysed by LC–MS, against a calibration curve of PMD in acetonitrile. As a control, the same copolymer was tested in the absence of PLEs. All experiments were performed in triplicate.

#### 2.2.7. In Vitro Hydrolysis of Monomer Drug Conjugate (APMD) by PLEs

The monomer drug conjugate (APMD) was dissolved in acetonitrile, followed by the dropwise addition of water with continuous stirring until a clear solution was obtained with a final proportion of water/acetonitrile of 9:1 (*v/v*) and a monomer concentration of 1.2 mg/mL in 10 mL. The pH of the solution was adjusted to 7 by adding ammonium acetate buffer before 54.3 mg of PLE (1 unit of enzyme per µM APMD) were added, and the solution was stirred at 32 ± 1 °C (corresponding to the skin temperature). Samples (0.75 mL each) were withdrawn periodically, quenched with an equivolume of acetonitrile (0.75 mL), centrifuged, and assayed as for the copolymer, with APMD in the absence of enzyme used as a control. All experiments were done in triplicate.

#### 2.2.8. In Vitro Hydrolysis with Enzyme Replenishment

To ensure that that the enzyme activity was maintained for extended times, the experiments described in Section 3.7 were modified to monitor long term in vitro hydrolysis of the copolymer. In brief, the protocol described above was modified with a fresh aliquot of enzyme added every 24 h.

#### 2.2.9. Skin Preparation

Fresh (non-steam-cleaned) pig ears obtained from a local slaughterhouse (within 6 h of animal sacrifice) were kept at −20 °C before membrane preparation; frozen skin was used within two months. Hair was trimmed before full-thickness membranes containing dermis and epidermis were harvested from the underlying cartilage using a scalpel.

#### 2.2.10. Skin Permeation and Uptake Studies

Porcine skin is a commonly employed alternative to human tissue in in vitro studies [3]. Full-thickness skin samples were mounted on glass Franz-type diffusion cells (*n* = 6 for each test), with diffusion areas of 3.14 cm^2^ and a receptor volume of 15 mL. Solutions (3 mg/mL) of PMD and the copolymer were prepared in water/ethanol (8:2 *v/v*) and 1 mL (3 mg) was dosed into the donor chamber. The Franz cells were placed in an incubator at 32 ± 1 °C; samples (1 mL) were taken periodically from the receptor compartment between 0.25 and 72.00 h, and were replaced with equivolume of the water/ethanol (8:2 *v/v*) receptor medium.

Following the 72 hrs permeation study, PMD and copolymer remaining in the tissue was assayed by tape stripping. The first two tape strips were assumed to account for analyte remaining on the skin surface, tape strips 3–10 accounted for deposition in the upper stratum corneum, and strips 11–20 showed analyte remaining in the lower stratum corneum. To extract the PMD or copolymer, tape strips were immersed in ethanol and sonicated for 15 min at room temperature. Any residual tissue was removed by filtration (0.45 μm filter, Thermo Fisher, United States), and the extraction liquid analysed by gel permeation chromatography (GPC) for the copolymer and LC-MS for PMD.

#### 2.2.11. Toxicity Testing Using a Planarian Fluorescent Assay

We have recently reported a novel planarian toxicity assay for early pre-screening of potential skin irritants [12] on the basis that irritants will disrupt the planarian outer membrane and allow a fluorescent dye to enter the animal. Planaria (*Dugesia lugubris*) were purchased from Blades Biological Ltd. (Kent, United Kingdom). Briefly, a planarian (pond worm) was exposed to 0.1% (*w/v*) of the test substance for 1 min, followed by washing with artificial pond water (APW) for a further 1 min. The planaria were then placed in a 0.1% (*w/v*) solution of sodium fluorescein in APW for 1 min. Finally, the planaria were washed with APW (15 mL) for 1 min to remove excess sodium fluorescein adsorbed to the outer worm surface. The test animal was then immobilized by embedding it in 2% agarose solution on a microscope slide, which was immediately placed on ice leading to gelling of the agarose solution and immobilisation of the test animal. Fluorescence images of individual planaria were collected with a Leica MZ10F stereomicroscope (Leica Microsystems, United Kingdom) with a Leica DFC3000G digital camera, 1.6× magnification with 160 ms exposure time (gain = 2.6×), Gamma = 0.7, and wavelength = 519 nm (excitation wavelength). The negative controls were planaria treated only with sodium fluorescein. Fluorescence of the whole animal was measured using ImageJ (version 1.8.0_112) software, and the value obtained normalised by dividing by the area (cm^2^) of the individual planarian. All experiments were done in triplicate.

## 3. Results and Discussion

### 3.1. Synthesis of Monomer Drug Conjugate (APMD)

Acryloyl chloride was selected to conjugate PMD via ester bond formation, and the resulting conjugated monomer (APMD) can be co-polymerised with acrylic acid; poly(acrylic acid)-based polymers are commonly used in topical drug delivery, and have an excellent safety profile. The chemical structure of APMD was verified by ^1^H NMR (see Supplementary Materials Figure S1), evidenced by the loss of the OH groups from PMD (δ 4.42 ppm) due to the formation of an ester bond between the PMD and the acryloyl chloride and the presence of CH_2_ and CH groups from acryloyl chloride at ~δ 6 ppm in the conjugated monomer. Furthermore, mass spectrometry confirmed a mass of 226 Da, in agreement with the anticipated mass of the C_13_H_22_O_3_ conjugate.

### 3.2. Synthesis of Poly(AA-co-APMD)

**Figure 1:**
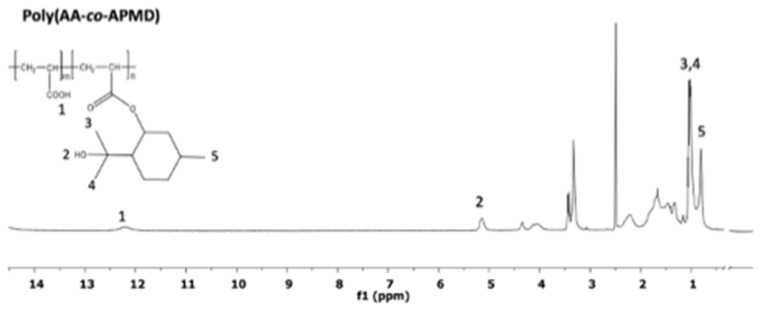
^1^H-NMR spectrum of poly(AA–*co*–acryloyl–p-menthane-3,8-diol (APMD)), showing resonances at (1) 12.25 ppm, (2) 5.2 ppm, (3) 1.2 ppm, (4) 1.1 ppm, and (5) 0.9 ppm.

The APMD conjugated monomer was copolymerised with acrylic acid to prepare a series of copolymers: poly(AA-*co*-APMD). These copolymers were synthesised by free radical polymerization, using AIBN as a free radical initiator. The ^1^H NMR spectra confirmed successful synthesis (Figure 1)—notably, the loss of resonance from the double bonds of the acryloyl moiety of APMD. Furthermore, the ^1^H NMR peaks broadened, a common phenomenon observed with polymers [13]. Infrared (IR) spectroscopy confirmed the presence of the ester bond in both the APMD monomer and the subsequent copolymer (see Supplementary Materials Figure S2), which is essential for enzymatically triggered PMD release.

### 3.3. Copolymer Composition and Monomer Reactivity Ratios

**Figure 2:**
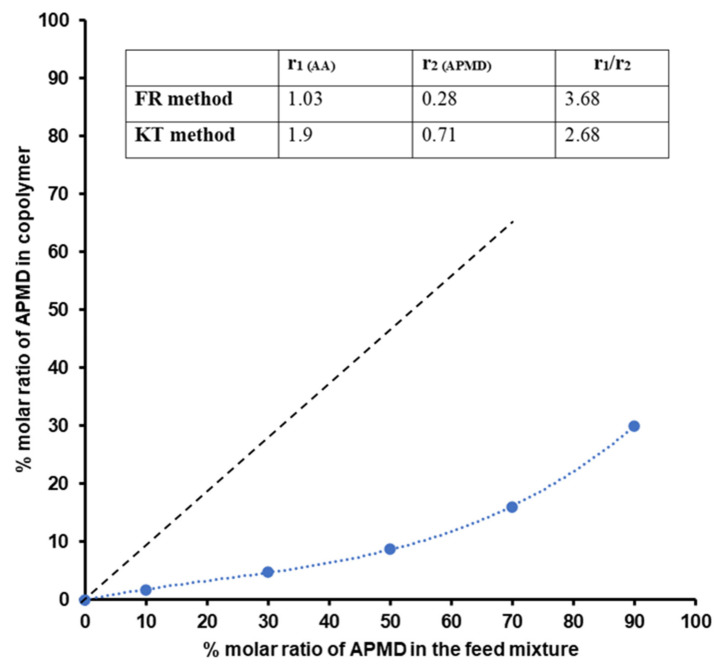
Composition of the copolymers as a function of APMD in the feed mixture. The dashed black line indicates the expected composition if the reactivity of the monomers was equivalent. Inset: reactivity ratios of acrylic acid (AA) and APMD, determined by the Finemann–Ross (FR) and Kelen–Tüdós (KT) methods.

Researchers commonly employ ^1^H NMR to quantify components in copolymers. Attempts to integrate the backbone versus the OH peak, or the ester-neighbouring CH_2_ peak, to calculate the ratio of incorporated units of each monomer were unreliable; protons in OH groups are exchangeable in protic solvents, and the presence of trace amounts of water in DMSO may confound accurate determinations. In the absence of a chromophore, an LCMS method was developed to assay unreacted AA and APMD (calibration curve available in Supplementary Materials, Figure S3). The results (Figure 2) show that the PMD-conjugated monomer, APMD, is less reactive than acrylic acid in free radical polymerisation. Consequently, the synthesised copolymer is not in the same molar ratio as the monomers in the feed mixture, which impacts the repellent loading in the system. As the proportion of APMD increases to 90% of the feed, up to 30% of the APMD can be included in the copolymer. However, for this composition, yields were very low (~2%), and so the better-yielding AA/APMD (30:70) system, which resulted in 16% APMD in the copolymer, was selected as the lead conjugate system. Furthermore, PMD constitutes 72% of APMD with the remainder the acryloyl group. Thus, the PMD that is available to be released from the AA/APMD (30:70) is approximately 11.5% of the total copolymer mass.

Given the large disparity in monomer inclusion in the copolymer, reactivity ratios (*r* = the ratio of the monomers in copolymerisation [14]) were calculated by application of conventional linearization methods, namely the Finemann–Ross (FR) and Kelen–Tüdós (KT) methods [15]. The results are summarised in Figure 2, with more details of the approach and plots available in the Supplementary Materials (Tables S1 and S2, Figures S4 and S5). While there are differences between the two methods, it is clear that AA is approximately three times more reactive than APMD, and hence it is expected that copolymers will contain a greater proportion of AA, and lower levels of the repellent carrying APMD, than anticipated simply from the feed composition. This finding is in agreement with previous literature showing that, as a monomer becomes bulkier, its reactivity tends to decrease; for example, when acrylic acid was copolymerised with methyl methacrylate, the reactivity ratio of the acrylic acid was 1.50, and for the bulkier methyl methacrylate it was 0.48 [14].

### 3.4. Molecular Weight Characterisation

GPC analysis demonstrated that the average molecular weight of the poly(AA–*co*–APMD) copolymers increased with increasing amounts of acrylic acid in the feed mixture (and consequent copolymer). This is expected, and agrees with the data showing that AA was more reactive than APMD, since monomers with higher reactivity tend to produce higher-molecular-weight polymers [16]. Furthermore, bulky cyclic groups are known to terminate polymerisations and decrease kinetic chain lengths [17], resulting in shorter polymers, as seen here when the proportion of APMD in the feed is high (Table 1). The polydispersity index for the polymers (Mw/number­–average molecular weight (Mn)) ranges from 2.1 to 3.4, within typical ranges for free radical polymerization, but could be controlled by the terminating the polymerisation reaction at low conversion rates [18]. With the low synthetic yield of the AA/APMD (10:90) system, the copolymer prepared using a 30:70 monomer feed ratio was selected for skin permeation testing, and provided a system with a suitable repellent loading and with a molecular weight over 10-fold greater (6200 Da) than the notional upper molecular weight limit (~500 Da) for permeants to readily enter and diffuse through skin.

### 3.5. Thermal Analysis

**Figure 3:**
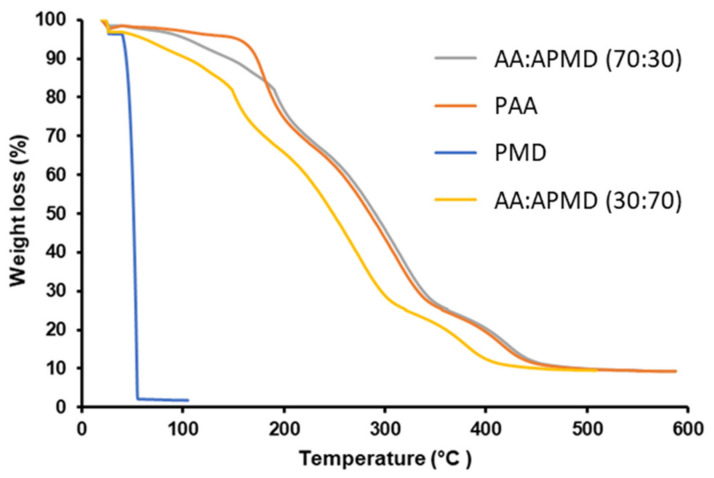
Thermogravimetric analysis of PMD and PAA (controls) and AA/APMD copolymers.

The thermal stability of the copolymers was explored and compared to that of PMD alone and poly(acrylic acid) (PAA) as a control. PMD is highly volatile and, when heated during thermogravimetric analysis, it evaporated between 45 and 60 °C. PAA is a widely used and suitably stable polymer, which showed minimal weight loss (attributed to surface adsorbed water) up to ~170 °C. Beyond this, the polymer starts to degrade, in agreement with the literature [19]. The presence of chemically-linked PMD in the copolymers prevented the rapid loss of weight seen from this terpenoid alone (Figure 3). For example, with the AA/APMD (30:70) copolymer, approximately 10% of the sample weight (assumed to be PMD) was lost on heating from 60 to 100 °C, in agreement with the data above (Section 3.3), where the 30:70 polymer composition indicated that PMD constituted approximately 11.5% of the total polymer weight. In contrast, for the AA/APMD (70:30) copolymer, approximately 3% of the weight was lost over the same range, indicative of the lower PMD loading in the system.

DSC analysis was performed on PAA alone and copolymers to determine the glass transition temperature (*Tg*). For 5000 Da PAA, the *Tg* was 101 °C, which is in agreement with the literature [20], while poly(AA-*co*-APMD) showed *Tg* values varying from 48–59 °C for different molar ratios. A trend was seen whereby increasing the proportion of AA in the copolymer increased *Tg*, i.e., for the AA/APMD (30:70) copolymer, the *Tg* onset was at 48.2 °C, whereas for the AA/APMD (70:30) system, it was seen at 51.9 °C. The higher polarity of AA, compared to APMD, allows increased dipole interactions between the molecules, and hence PAA has rigid chains and a higher *Tg*. The addition of increasing amounts of the APMD repeating units decreases the polarity and the dipole interactions, leading to decreased intermolecular interactions, which ultimately makes the structure less rigid.

### 3.6. Analysis of PMD

Developing a suitably sensitive and accurate assay from PMD was problematic. The volatile chemical does not possess a chromophore, and HPLC or UV methods were impractical. A gas chromatography–mass spectroscopy (GCMS) method has been reported in the literature [21], but was not sufficiently sensitive for our work. Thus, liquid chromatography–mass spectroscopy (LC–MS) was used to analyse and quantify PMD.

Initial studies evaluated a C-4 column and a cyano (CN) column (columns with cyanopropyl groups) with 1% formic acid as the mobile phase, but resulted in excessive tailing and poor peak separation. A phenyl column with 2% formic acid gave better peak separation, and no significant tailing was found, as the column has better affinity for the PMD OH groups, thus leading to higher retention and better separation of the peak.

From the chromatogram, the peak at 155 Da was selected, correlating with the removal of one OH group from PMD, and provided a retention time of 3.11 min. The calibration curve for this method is in the Supplementary Materials (Figure S6**)**. The limit of detection (LOD) for PMD was determined to be 1 μg/mL, with a limit of quantification (LOQ) of 5 μg/mL. Inter-day (94.3%) and intra-day (91%) precision was assessed, and the linearity (*R*^2^) of the method was 0.9986; while the ICH guidelines require an analytical method to have a linearity (*R*^2^) of >0.999 with an accuracy of 100% ± 2%, as well as an intra- and inter-day precision of ≤2% (as residual standard deviation). The assay developed was suitably accurate and reliable to assay PMD release from our copolymer.

### 3.7. In Vitro Hydrolysis of the Copolymer

**Figure 4:**
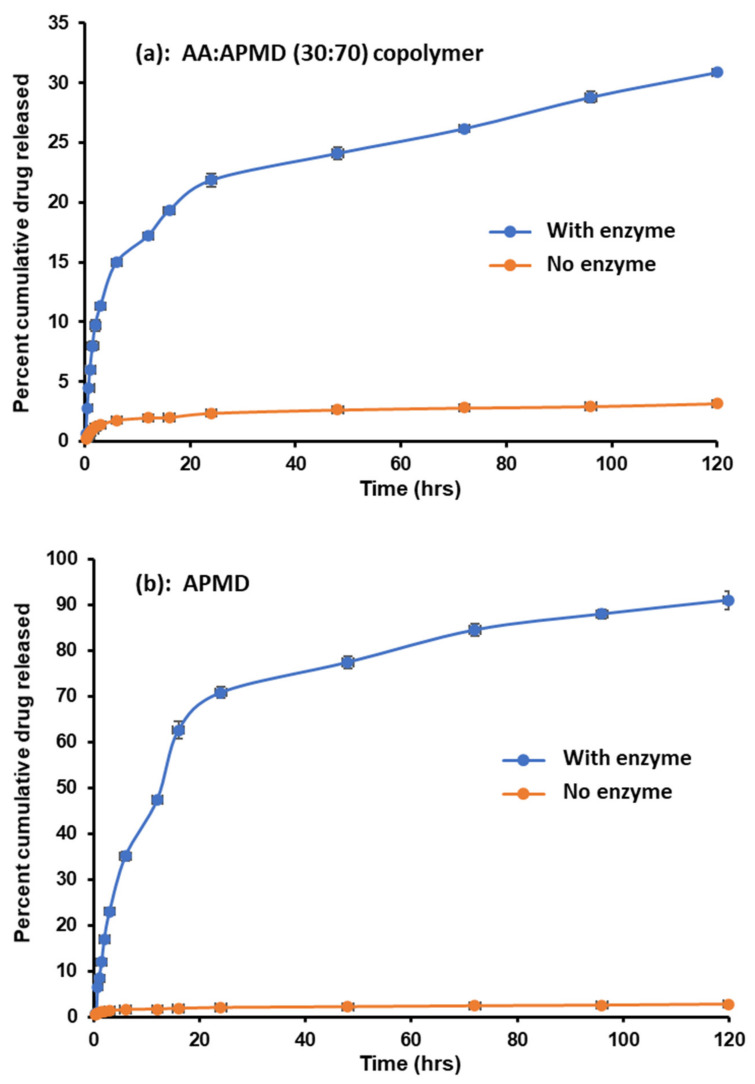
Release of PMD from (**a**) the AA/APMD (30:70) copolymer, with or without porcine liver esterases, and (**b**) from the monomer–repellent conjugate (APMD). Data are mean ± SD (*n* = 3).

Hydrolysis of the ester bonds was investigated by incubating the copolymer samples with PLEs to confirm that it was indeed a substrate for the enzyme. Thus, the AA/APMD (30:70) copolymer and PLEs were incubated at 32 ± 1 °C (to mimic the skin surface temperature), with a control of the copolymer incubated without PLEs to evaluate the stability of the ester bond. Experiments used 50 mg of the copolymer, previously shown to contain 11.5% PMD, and so the data below are expressed relative to the PMD content of 5.75 mg. PMD release from the copolymer in the presence and absence of the enzymes is shown in Figure 4a.

The results show significant hydrolytic release of PMD from the copolymer, with relatively little release seen in the absence of the enzymes. Initial enzyme-mediated release was rapid, with almost half of the PMD liberated over the first 6 h of incubation, followed by slower but linear release from 24 h to 5 days of incubation, such that, at the end of the experiment, 31% of the PMD was liberated (1.78 mg). With the control experiment and no enzymes, a small initial burst of PMD was also evident in the first 6 h, but only ~1.7% of the loaded repellent was liberated. This is again followed by a period of slow but sustained release with a total of 3.1%, equivalent to 0.18 mg, of PMD released after 5 days, i.e., an order of magnitude less than when the enzymes are available.

Release from the polymeric backbone is governed by multiple factors, including molecular weight, hydration of a polymeric prodrug, steric hindrance, and distribution of the drug along the polymeric chain [22]. It has been reported that drug release rates decrease with increasing molecular weight, due to greater chain entanglements that impede the diffusion of drug molecules (or here also, the enzymes) through the polymer matrix [23]. Likewise, increasing the extent of hydration (or polymer hydrophilicity) increases drug release [24]. In addition, steric hindrance can restrict enzyme access to the ester bond [25,26], and the distribution of a drug along the polymeric chain also plays an important role; pendant drugs distributed uniformly along the polymer backbone release faster than those that have them in blocks [27].

In order to test the above factors, we evaluated PMD release from the monomer–repellent conjugate (i.e., APMD) to determine if steric constraints or chain entanglement restricted release from the copolymer. Furthermore, the activity of the enzymes over extended periods was assured by replenishing the enzymes every 24 h [28].

Enzyme replenishment had no effect on PMD release from the copolymer. In the initial study, after 5 days incubation with PLEs, 1.78 mg of PMD was released, whereas when fresh enzyme was added daily, 1.80 mg PMD was released. However, PMD release from the monomer drug conjugate was markedly greater than from the copolymer (Figure 4b); over 90% of the total repellent was released over 5 days. It should be noted that the total PMD content in these samples (without the AA of the copolymer) was higher than for the copolymer at 8.7 mg, but the enzyme activity was adjusted accordingly. Again, an initial burst phase was seen, extending to 24 h, by which time nearly 80% of the PMD had been liberated. In the absence of enzymes, release from the monomer–repellent conjugate was again modest: 2.7% of the total PMD released after 5 days, which was not significantly different (*p* < 0.05) from the 3.1% released in the control experiment with the copolymer over this period. These results clearly indicate that the copolymer molecular weight influences repellent release, probably due to chain entanglements and steric hinderance that hampers diffusion of the enzymes through the polymer matrix and inhibits access of the enzymes to the ester bonds, leading to a decrease in PMD release compared to the free monomer conjugate [23]. Furthermore, the kinetics of initial PMD release from the copolymer (first 2 h) was assessed. Zero order, first order, Higuchi, and Korsmeyer–Peppas [29] mechanisms were tested, and while a good fit was found to the zero-order model, the data best correlated (*R*^2^ = 1.00) with the Higuchi square root of time kinetics, indicating diffusion-controlled release that was in agreement with the above observations for PMD liberated from the copolymer and monomer conjugate (see Supplementary Materials Figures S7– S10, Table S3).

### 3.8. In Vitro Skin Penetration and Permeation

Excised porcine skin (full-thickness ears) was used for permeation and uptake studies of PMD alone and the AA/APMD (30:70) copolymer (*n* = 6). Since both copolymer and PMD are very sparingly soluble in water, water/ethanol (80%:20% *v/v*) donor and receptor solutions were used.

**Figure 5:**
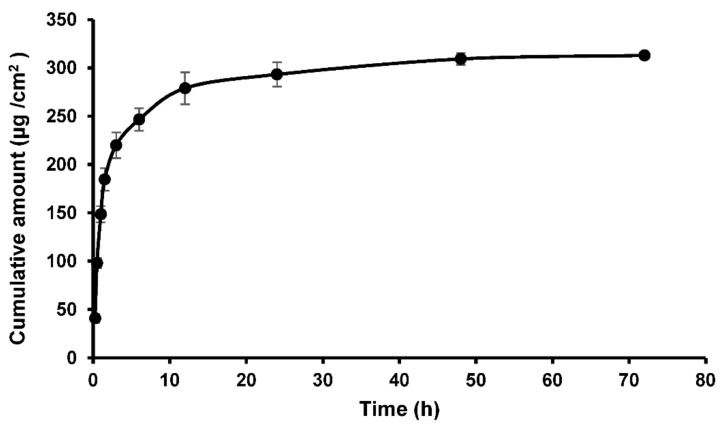
Permeation of PMD through full-thickness porcine skin; permeation of the copolymer was not detected at any time point. Data are mean ± SD (*n* = 6); there error bars are within the symbol where not seen.

When applied as a finite dose, PMD provided a typical permeation profile, shown in Figure 5, with rapid initial permeation before the donor depletion effects were seen. Over the first 6 h, 246 μg/cm^2^ permeated, and by 72 h a total 0.313 mg/cm^2^ had diffused through the tissue. With a diffusional area of 3.14 cm^2^, a total mass of 0.983 mg PMD had been delivered through the skin sample, equating to 32.8% of the applied dose (3 mg). In contrast, when dosed with the conjugate, no permeant was detected in the receptor fluid; both intact conjugate and PMD were sought, but there was no evidence that PMD released and permeated from the conjugate. It was anticipated that some residual esterases within the porcine skin would remain active and cleave the conjugate, thus allowing permeation of the repellent. It is feasible that repellent was released but volatilised rather than permeated, or that permeation was below the level that could be detected in our assay.

**Figure 6:**
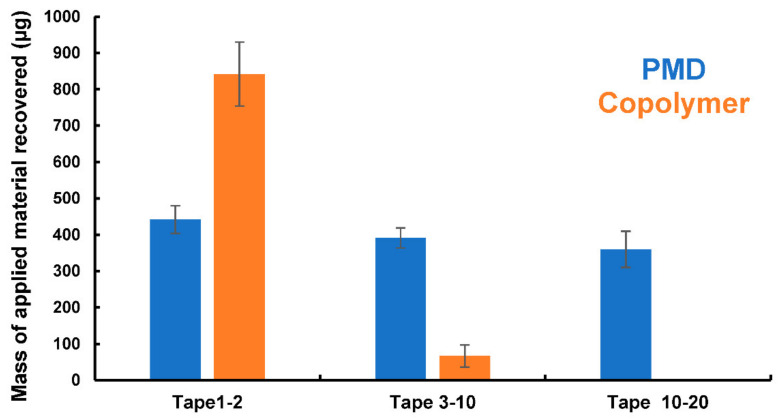
Skin uptake of PMD (control) and AA/APMD (30:70) copolymer in full-thickness porcine skin. Data presented as mean ± SD (*n* = 6).

Following the permeation study, PMD and conjugate within the tissue was assayed by tape stripping. The analytes were extracted into ethanol from the tape strips, and needed to be aggregated to allow accurate determination. Thus, tapes strips 1 and 2 represents the surface-adsorbed materials, tapes 3–10 were aggregated to account for analyte in the outer stratum corneum of the skin, and strips 11–20 aggregated to quantify the material in the lower stratum corneum. Figure 6 shows that when PMD was applied, the repellent was distributed throughout the stratum corneum at the end of the permeation study. Of the total PMD detected, 37% was in the superficially adsorbed sample (strips 1–2), 33% was in strips 3–10, and 30% was in strips 11–20. Near-uniform distribution of PMD in the upper and lower stratum corneum implies that the PMD had, in fact, reached near-steady state distribution throughout the stratum corneum after 72 h exposure. In terms of mass balance, the total PMD detected in the stratum corneum, plus that which permeated, was 79% of the applied dose, but repellent within the viable epidermis was not quantified. In addition, although our Franz diffusion cells were covered, PMD is highly volatile, and so some vaporisation of the repellent may have occurred, and some material may have volatilised during tape stripping and analyte recovery. In contrast, with the copolymer, the majority of the material remained at the skin surface in tapes 1–2, approximately 93% of the applied dose. The remaining 7% was detected within the outer stratum corneum tape strips (3–10), with no material detected deeper into tissue (tapes 11–20). Again, no liberated PMD from the conjugate was found in the tape strips.

### 3.9. Toxicity Testing of the Copolymer

A rapid assay was used to assess the potential toxicity of the copolymer. Planaria were exposed to the potential irritants, and then uptake of a fluorescent dye into the animal measured; chemicals that damage the worms’ outer membrane are potential irritants, and the fluorescence intensity within the worm after treatment has been correlated with skin irritation tests on higher vertebrates, such as the primary irritation index [12]. Here, PAA, PMD, and the AA/APMD (30:70) copolymer were tested against benzalkonium chloride (BKC) as a positive irritant control, while artificial pond water (APW) with and without DMSO (1% *v/v*) was used as the negative control for water-insoluble and water-soluble compounds, respectively.

**Figure 7:**
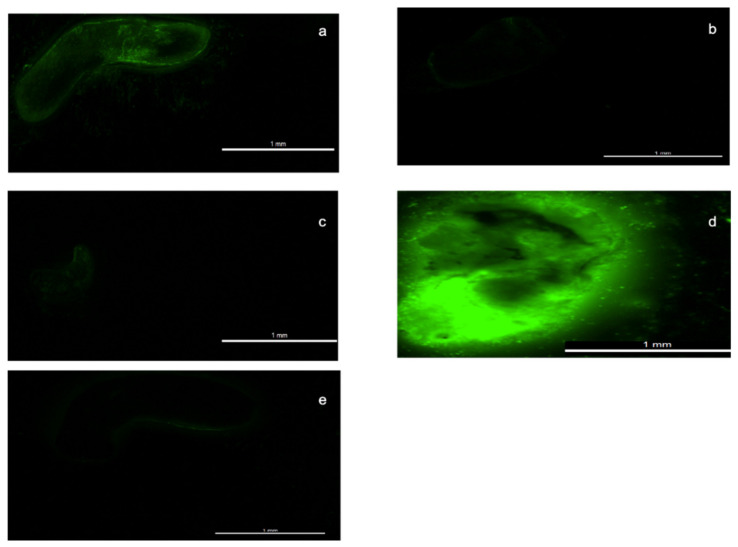
Exemplar fluorescent images of artificial pond water (APW) with fluorescein in DMSO (**e**), and after planaria were exposed to PAA (**a**), PMD (**b**), copolymer (**c**), and benzalkonium chloride (BKC) (**d**).

The fluorescence intensity (FI) of planaria exposed to artificial pond water before immersion in the fluorescent dye (negative control) was minimal, and indistinguishable from background fluorescence showing that the planarian membranes were intact. FI values were 2.6 and 2.5 arbitrary units (a.u.) after treatment with APW with or without 1% DMSO, respectively, in accordance with our previous report [12]. With the positive irritant control, BKC, FI was 52.6, again in good agreement with the earlier study and showing the strong interaction of this irritant in destabilising the worm membrane, allowing the fluorescent dye to enter (Figure 7).

According to the Globally Harmonized System of Classification and Labelling of Chemicals (GHS), PMD can cause eye damage and irritation, but is not listed as a skin irritant. The United States Environmental Protection Agency has approved the use of PMD-containing insect repellents, and placed PMD into Toxicity Category IV (very low toxicity) for acute oral toxicity, dermal toxicity, and skin irritation, but in Toxicity Category I for eye irritation. It is not a skin sensitizer, and there were no observed adverse effects from a 90-day dermal toxicity study in rats up to a dose of 1000 mg/kg/day. As expected, the results of our planarian assay showed PMD to be a non-irritant with a FI of 4.3 a.u. after exposure to 0.1% PMD.

**Figure 8:**
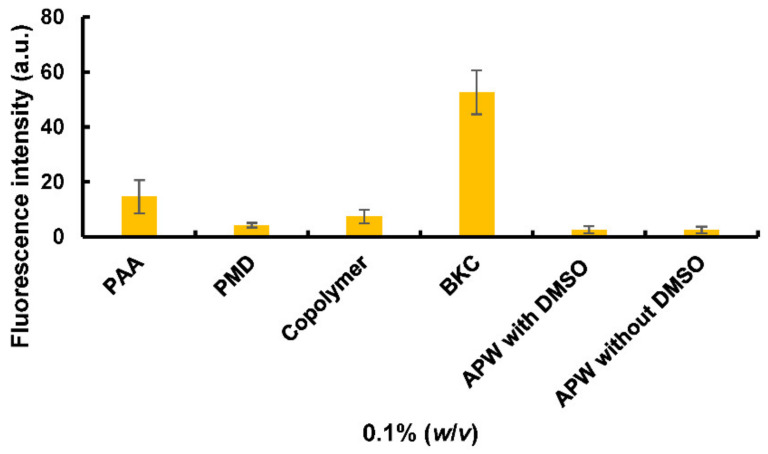
Fluorescence intensity (per cm^2^) of individual planaria exposed to different test substances. Data are expressed as mean ± standard deviation (*n* = 3).

While high-molecular-weight, weakly cross-linked poly(acrylic acid) is widely used in topical products, and has a long history of safe use, low-molecular-weight PAA can be an irritant. For our 6000 Da material, the FI of planaria treated with PAA was 14.6 a.u., similar to values obtained with other mild irritants [12]. However, in the poly(AA–*co*–APMD) copolymer, the FI value fell to 7.5 a.u., indicating a suitable safety profile that merits further regulation-approved dermal toxicity and irritation testing (Figure 8).

## 4. Conclusions

Free radical polymerisation of AA and APMD allowed the formation of poly(AA-*co*-APMD) ester copolymers with low to medium molecular weights (dependent on the feed monomer ratios). The proportion of the APMD in the copolymers was lower than expected due to the greater reactivity of AA. After verifying the stability of the volatile repellent in the copolymers, the AA/APMD (30:70) copolymer containing approximately 11.5% PMD was further evaluated. Incubation with porcine liver esterases demonstrated that the ester bond could be cleaved to liberate the repellent over extended periods. However, release was partial when compared with release from the “free” monomer, demonstrating some hindered access for the enzymes to the ester bonds, consistent with diffusion-controlled kinetics. Whereas free PMD was able to penetrate into and permeate through porcine skin, no permeation of the copolymer with conjugated PMD through the tissue was detected, and the polymer was principally confined to the outer surface of the skin. Finally, a simple fluorescence assay suggests that the copolymer is likely to have similar or lower skin irritation than the equivalent molecular weight poly(acrylic acid). Our study demonstrates a novel use for drug (repellent)–polymer conjugates in retaining active ingredients on the skin surface, reducing uptake into and permeation through the skin, and exploiting endogenous enzyme systems to provide extended release of the volatile active ingredient over extended periods of time.

## Figures and Tables

**Table 1:** Molecular weights of poly(AA–*co*–PMD) prepared from different feed compositions.

Feed Molar Ratio (AA/APMD)	Mw	Mw/Mn
10:90	1800	2.1
30:70	6200	2.9
50:50	11,500	3.1
70:30	29,200	3.3
90:10	53,100	3.4
Poly(acrylic acid) (PAA) (100:0)	252,000	3.4

Mw = weight–average molecular weight; Mn = number–average molecular weight.

## Supplementary Materials

The following are available online at https://www.mdpi.com/1999-4923/13/3/403/s1, Figure S1: ^1^H-NMR data showing conjugation of PMD to acryloyl chloride to form APMD. Figure S2: IR spectra showing presence of an ester link in the poly(AA-*co*-APMD). Figure S3: LC–MS calibration curve to quantify APMD. Figure S4: Plot for determining monomer reactivity ratios in the copolymerisation of AA and APMD by the FR method. Figure S5: Plot for determining monomer reactivity ratios in the copolymerisation of AA and APMD by the KT method. Figure S6: LCMS Calibration curve for PMD. Figure S7: Zero order PMD release plot. Figure S8: First order PMD release plot. Figure S9: Higuchi (square root of time) PMD release plot. Figure S10: Korsmeyer-Peppas PMD release plot. Table S1: Compositional parameters for poly(AA-*co*-APMD) reactivity ratios by the FR method. Table S2: Compositional parameters for poly(AA-*co*-APMD) reactivity ratios by the KT method. Table S3: The correlation coefficients and release rate constants for PMD release from poly(AA-*co*-APMD).
