# Supplementary material for: A Novel Polymer Insect Repellent Conjugate for Extended Release and Decreased Skin Permeation of Para-Menthane-3,8-Diol
Source: Pharmaceutics. 2021 Mar 18;13(3):403. doi: 10.3390/pharmaceutics13030403 (PMC8002994; doi:10.3390/pharmaceutics13030403)
Supplement: Supplementary file 1 [file pharmaceutics-13-00403-s001.pdf]

pharmaceutics

Supplementary Materials: A Novel Polymer Insect Repellent Conjugate for Extended Release and Decreased Skin Permeation of Para-Menthane-3,8-Diol

Syed I Shah, Vitaliy V Khutoryanskiy and Adrian C Williams *

Acryloyl chloride

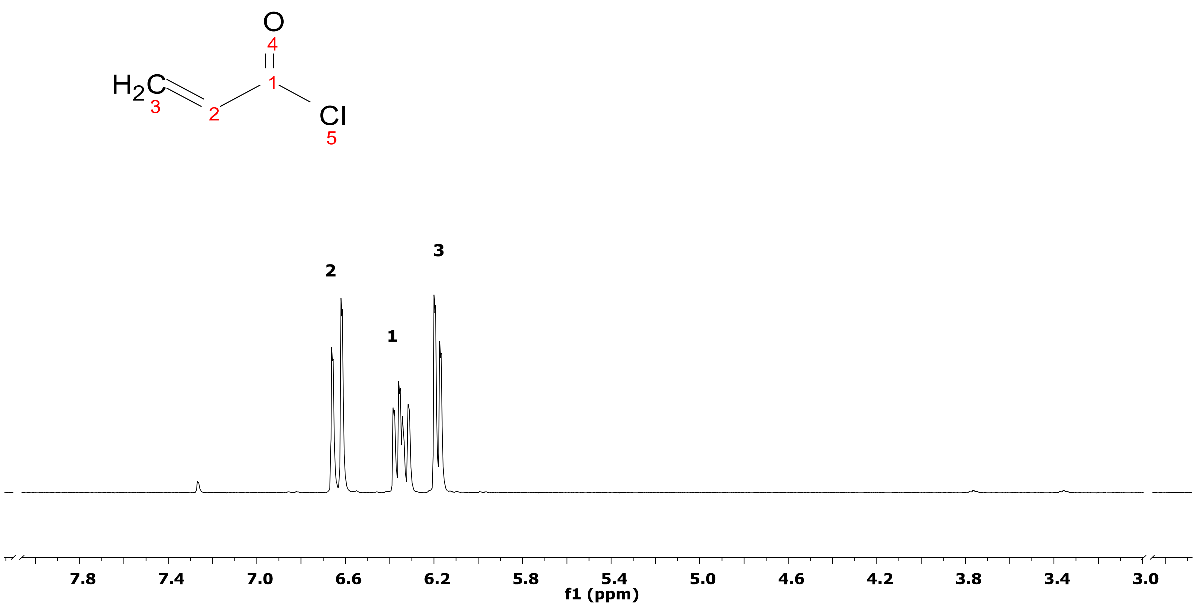

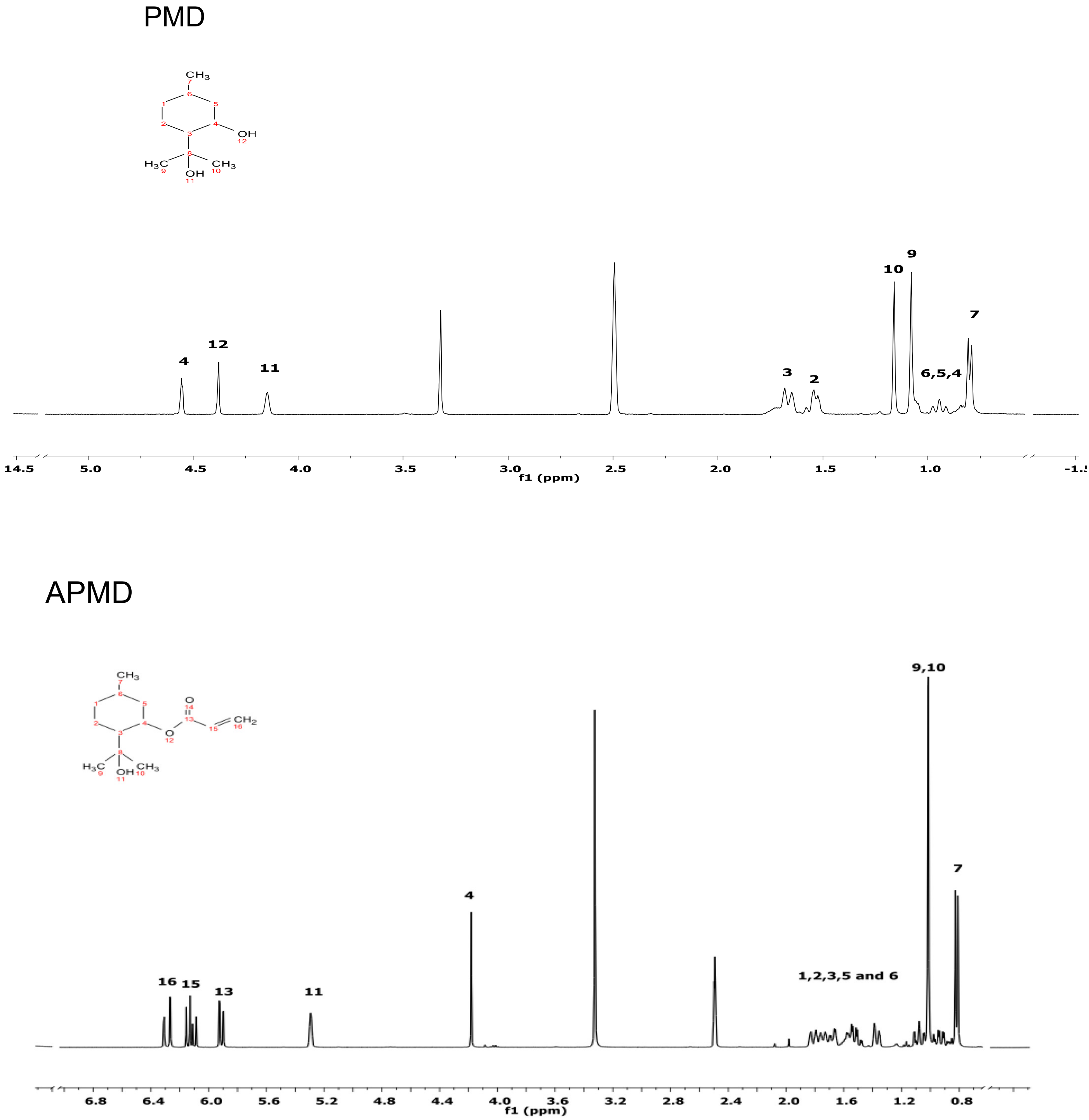

Figure S1. ^{1}H-NMR spectra of the starting material (acryloyl chloride and PMD) and the monomer conjugate (APMD) showing successful conjugation between PMD and acryloyl chloride.

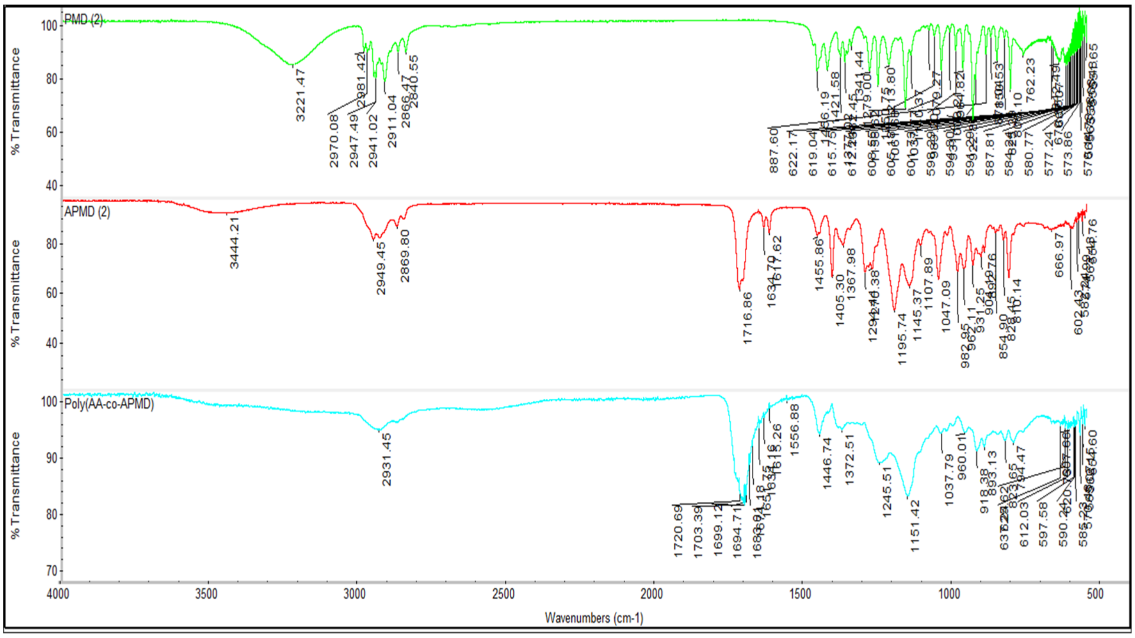

Figure S2. IR spectra showing TOP: PMD with characteristic peaks for OH (3217 cm^{-1}), CH stretching (2840-2970 cm^{-1} region); MIDDLE: APMD showing a prominent peak at 1716 cm^{-1} indicating formation of ester bond: BOTTOM: the co-polymer, poly(AA-*co*-APMD).

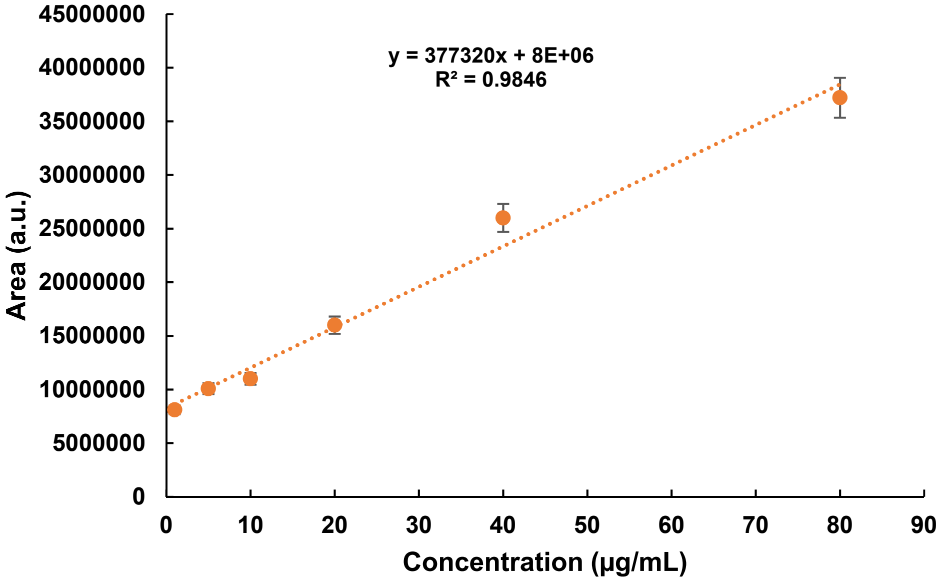

Figure S3. LC–MS calibration curve to quantify APMD. Data are mean ± SD, *n* = 3.

Determination of reactivity ratios

The Finemann–Ross (FR) method is commonly used for determining reactivity ratios, where G and H (numerical values obtained through the initial feed and final copolymer) are related according to:

$$G = r_{APMD}H - r_{AA} \quad (1)$$

The FR plot obtained by linear regression analysis for AA/APMD copolymers is in Figure S4 using the parameters summarised in Table S1.

Table S1. Compositional FR Parameters for Poly(AA-*co*-APMD) Copolymer System.

F	f	f^2/F (H)	f(1−F/f) (G)
0.11	0.063	0.036	0.5
0.42	0.176	0.073	0.24
1	0.449	0.201	0
2.33	1.38	0.81	0.78
9	8.09	7.27	7.19

F = %$_{APMD}$ in monomer mixture/total %$_{APMD}$, i.e. 1/9=0.11, 3/7 = 0.42, and f = %$_{APMD}$ in copolymer mixture/total %$_{APMD}$, i.e. 0.6/0.94 = 0.063, 0.15/0.85 = 0.176.

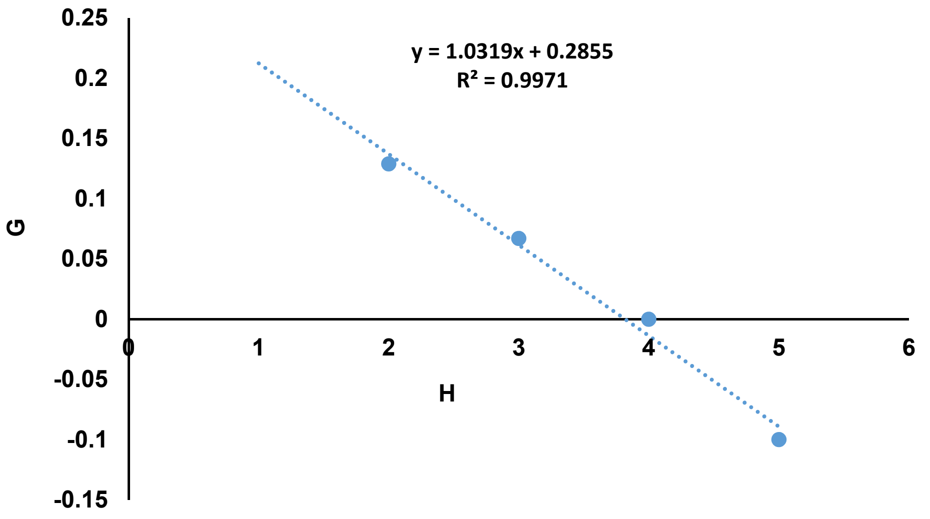

Figure S4. FR method for determining monomer reactivity ratios in the copolymerisation of AA and APMD from LCMS data.

Kelen and Tüdós (KT) applied these two parameters, *i.e.* *G* and *H* in the linearized copolymerization equation, along with new parameters such as α, η and ζ. The intercepts at $\zeta = 0$ and $\zeta = 1$ of the η versus ζ plots yield – r_{APMD}/α and r_{AA}, respectively.

Table S2. Compositional KT Parameters for Poly(AA-*co*-APMD) Copolymer System.

F	f	f^2/F (H)	f(1−F/f) (G)	η	Ξ
0.11	0.063	0.036	0.5	0.89	0.06
0.42	0.176	0.073	0.24	0.40	0.12
1	0.449	0.201	0	0	0.27
2.33	1.38	0.81	−0.78	−0.58	0.60
9	8.09	7.27	−7.19	−0.92	0.93

$\alpha = 0.52$, $\eta = G/(\alpha + H)$ and $\xi = H(\alpha + H)$.

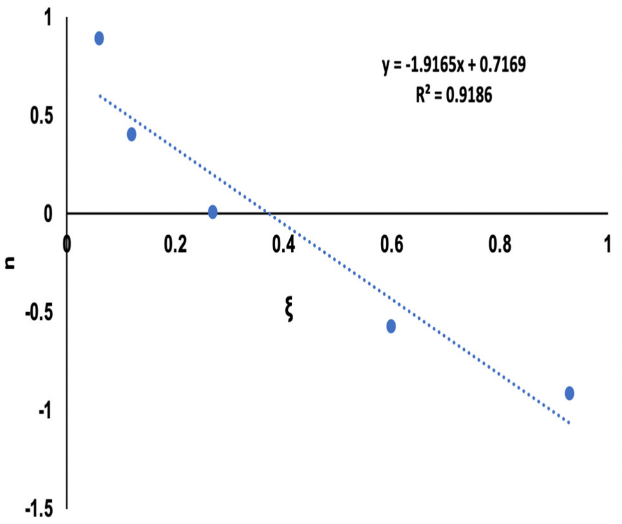

Figure S5. KT method for determining monomer reactivity ratios in the copolymerisation of AA and APMD from LCMS data.

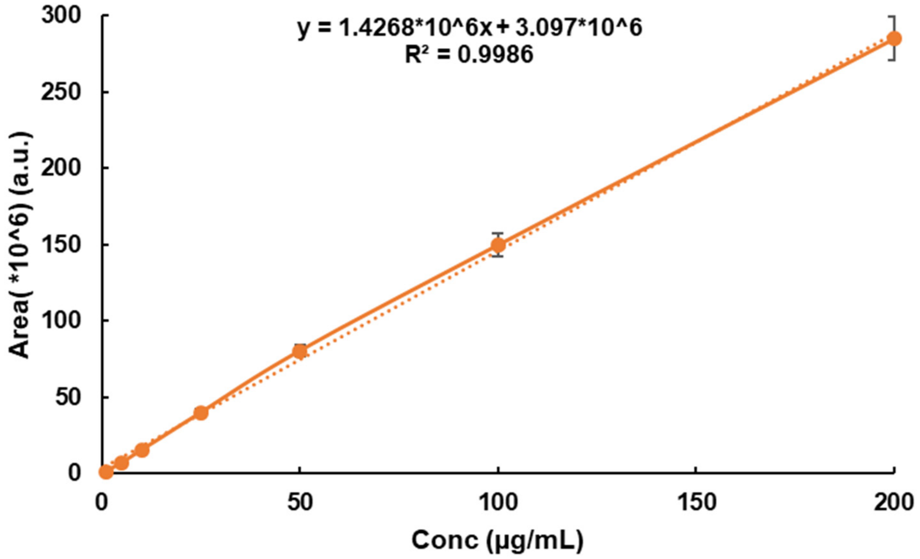

Figure S6. PMD assay development. LCMS Calibration curve for PMD using the peak at 155 Da, due to the loss of one -OH group. Data are mean ± standard deviation (n = 3).

To study the kinetics of the drug release, initial 2 h drug release data was used. The release constants were calculated from the slope of appropriate plots, and the correlation coefficient (r^2) by linear regression analysis using Microsoft Excel 2016. The correlation coefficient was selected to evaluate the appropriate kinetic model and the corresponding release kinetic data for the synthesized copolymer, though it is appreciated that several mechanisms may operate concurrently. Both zero order (r^2 = 0.98) and square root of time / Higuchi models (r^2 = 1.00) gave strong fits

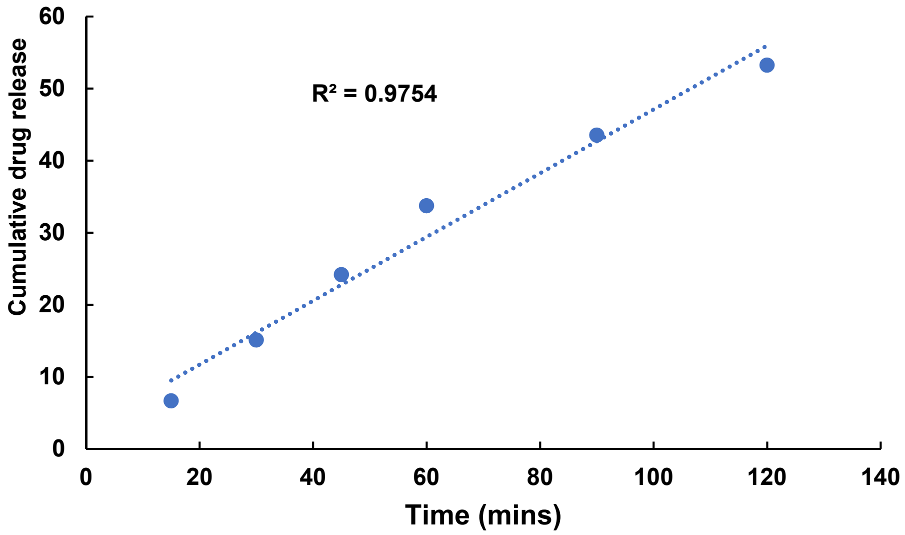

Figure S7. Zero order release plot (n = 3).

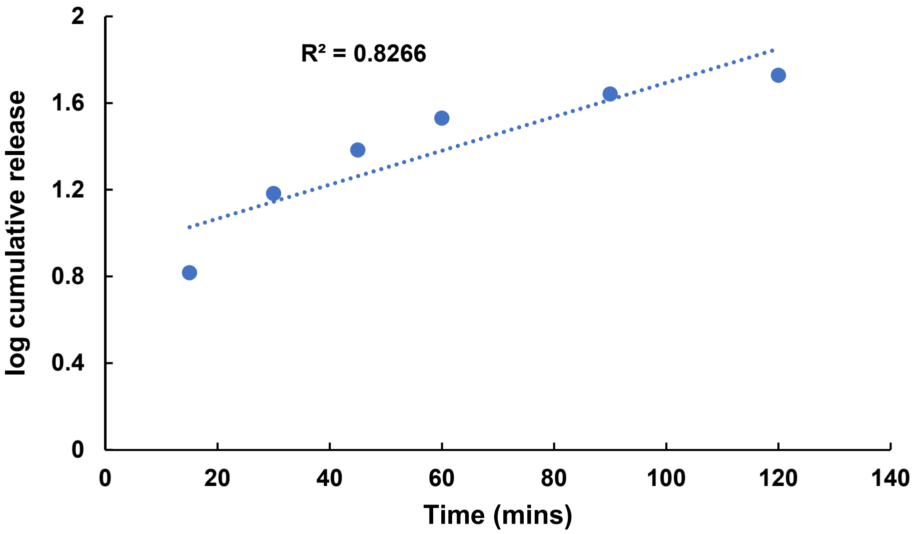

Figure S8. First order release plot (n=3).

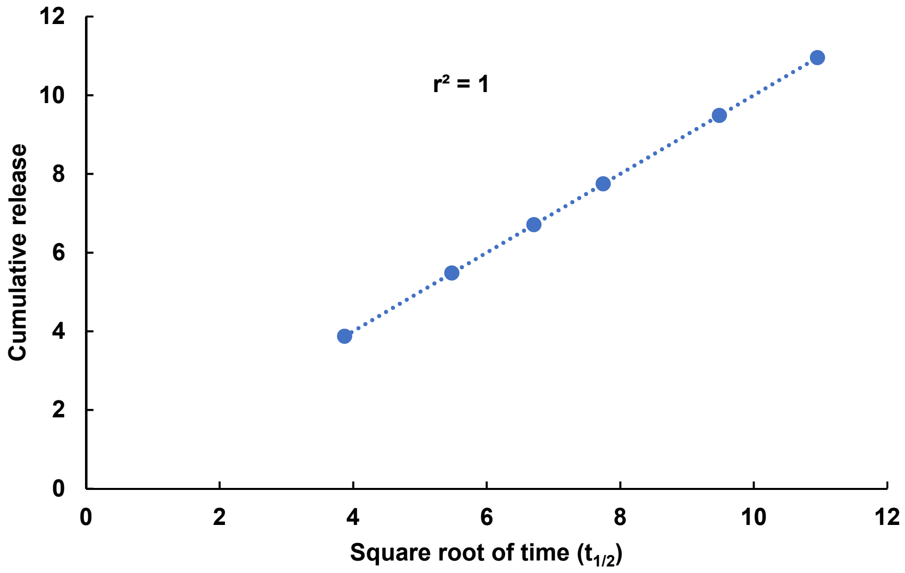

Figure S9. Higuchi (square root of time) release plot (*n*=3).

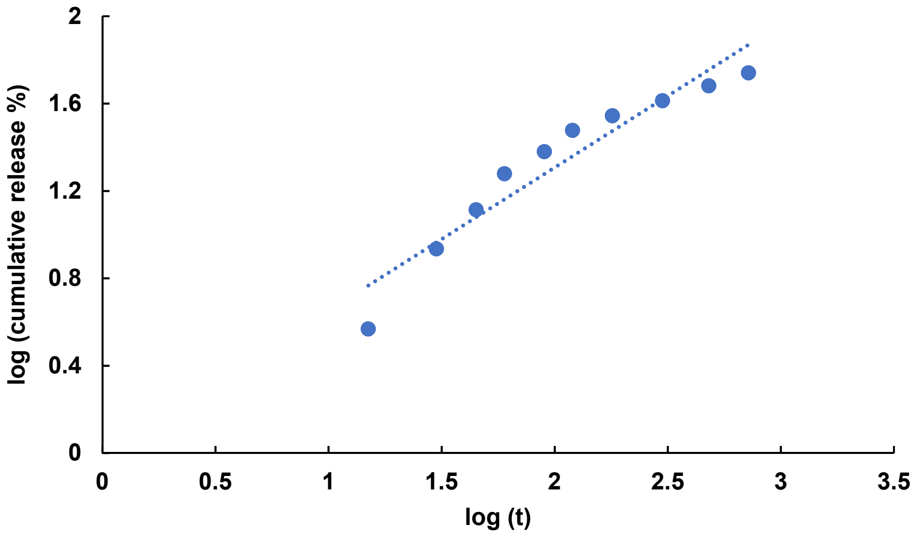

Figure S10. Korsmeyer-Peppas release plot (*n* = 3).

Table S3. The correlation coefficients and release rate constants for PMD release from poly(AA-*co*-APMD).

Drug Release Model	r^2 value	k value (h^{-1})
Zero order	0.97	0.4426
First order	0.82	0.0078
Higuchi's model	1.00	1.00
Korsmeyer-Peppas model	0.91	*n* = 0.655

References

C. Erbil, B. Terlan, Ö. Akdemir, and A. T. Gökçeören, "Monomer reactivity ratios of N-isopropylacrylamide–itaconic acid copolymers at low and high conversions," *Eur. Polym. J.*, vol. 45, no. 6, pp. 1728–1737, 2009.
